# Mentalization and Its Relation to Life Satisfaction and the Level of Mental Adjustment to Illness in Women with Breast Cancer—A Pilot Study

**DOI:** 10.3390/ijerph191610323

**Published:** 2022-08-19

**Authors:** Mariusz Cieślak, Joanna Kozaka, Paulina Beata Golińska, Mariola Bidzan

**Affiliations:** 1 Institute of Psychology, University of Lodz, 90-136 Lodz, Poland; 2 Private Counseling Services Joanna Kozaka, 80-414 Gdansk, Poland; 3 Department of Neuropsychology, University of Gdansk, 80-309 Gdansk, Poland; 4 Department of Clinical and Health Psychology, University of Gdansk, 80-309 Gdansk, Poland

**Keywords:** oncological diseases, psychological resources, mental health

## Abstract

This study’s aim was assessing of the relationship between mentalization and life satisfaction and the level of adaptation to oncological disease in patients with breast cancer. The study involved 41 women (*M* = 59.88; *SD* = 8.81) with breast cancer who completed their treatments and participated in a cancer rehabilitation program. In the study, we used the Mentalization Questionnaire (MZQ), the Satisfaction Life Scale (SWLS), and the Mini-Mental Adjustment to Cancer Scale (Mini-MAC). The mean of declared level of mentalization was 45.54 (*SD* = 11.65). The significant correlation between mentalization (its general value and individual dimensions) and satisfaction with life/mental adjustment to cancer was observed. The strongest positive correlations were noted between refusing self-reflection, helplessness–hopelessness, and anxious preoccupation and the negative correlation between mentalization and satisfaction with life. Mentalization was a predictor of satisfaction with life and mental adjustment to illness. Mentalization was related with life satisfaction and adjustment to cancer in patients with breast cancer, which is in line with previous studies suggesting the crucial role of subjective psychological factors in maintaining mental health.

## 1. Introduction

Despite significant development and progress in medical sciences related to cancer, the illness continues to provoke powerful reactions—primarily negative ones. A cancer diagnosis may trigger a crisis in a patient, particularly if it is sudden and unexpected. Over the past forty years, the number of new cancer diagnoses and related deaths has risen very sharply. In 2019, approximately 171,200 new cases of cancer and around 100,300 related deaths were recorded in Poland. Malignant tumors are the second major cause of death in Poland, leading to 25.7% of all deaths among men and 23.2% among women [1]. Young adult women (aged 20 to 44) are twice as likely to be diagnosed with cancer than men; this trend has remained unchanged from the early 1980s until today. In 2019, women were most frequently diagnosed with breast, lung, and endometrial cancer, and most deaths were caused primarily by lung and breast cancer. Among middle-aged women (aged 45 to 64), cancer is responsible for 50% of deaths [1]. Fatality rates increase with age and peak among patients in their seventies and eighties [2].

### 1.1. Cancer and Mentalization

Cancers are classified as diseases that constitute an immediate risk to life, additionally leading to adverse changes in many areas, such as biological, cognitive, emotional, and behavioral [3,4]. They may cause intrusive thoughts related to the illness, avoidant attitudes and behaviors, sleep and waking disorders, or attention deficits [5]. The lack of control and sense of danger often forces patients to reassess their previous ideas about health and sickness, who and what kind of person they are (i.e., a person “with cancer”), the people around them, the world they live in, and what the future has in store for them (premature death) [6].

Tackling a cancer diagnosis requires the engagement of psychological resources. Mental functioning with a cancer diagnosis mainly depends on one’s ability to comprehend, analyze, perceive, and process the situation [7]. One crucial aspect of functioning is the mentalization dimension (reflective function). Authors of the mentalization concept define it as “the ability to give meaning to one’s actions and the actions of other people through relating to intentional mental states, as well as the ability to process emotions and organize one’s own experience” [8,9]. The ability to mentalize also forms the basis for constructing a coherent and stable personality structure [10]. Mentalization cannot be seen on an either–or basis. It is described using dimensions that create continuums that determine the level of ability to keep a “mind inside a mind” [9]. Mentalization is one of the most mature and complex mechanisms that influence emotional regulation; it increases one’s capability to deal with complex and conflicted emotional states. All of this serves to increase one’s sense of security and agency and gives one inner direction and independence [8,9,10]. Cancer may involve experiencing numerous negative emotions in the form of pain and a lack of confidence. Chronic disease may be associated with long-term suffering and an arduous healing process. Cancer can often evoke thoughts of death in human consciousness, leading to feelings of hopelessness, helplessness, and depression.

This may reduce one’s ability to cope, resulting in a significant increase in the level of mental function and impacting numerous processes of analyzing the environment and internal emotional states experienced by patients. To deal with their situation, patients employ various coping mechanisms (i.e., denial, avoidance, repression, or escape into a world of dreams) [7,11].

As a result of their attempts to cope with the situation and intrusive ruminations, patients experience numerous negative emotional states. Usage of such mechanisms often leads to effects contrary to their intended ones. Patients may start to experience their situation anew [12]. This is why the best way of letting go of pain is to try to become aware of it: to mentalize it or, in other words, analyze it, process it, and make it part of one’s new identity [8]. Finding sense in pain increases the chance that experiencing it will lead to positive changes. As one of the primary mental processes, mentalization plays an important role in mechanisms for coping with various disorders. Therapeutic techniques involving mentalization are used in the treatment of PTSD, eating disorders, personality disorders, addictions, and for helping people going through a crisis [13].

Mentalization has rarely been a subject of research in Polish and foreign literature—particularly in clinical groups. The issue is worth examining as, in the case of cancer, how patients think about their illness and their attitude towards treatment impact the result of treatment [7].

### 1.2. Life Satisfaction and Adaptation in Cancer Patients

Life satisfaction, based on the state of one’s health, is strongly linked to one’s quality of life [14]. It constitutes a cognitive component of a person’s subjective well-being as it relates to the cognitive process as part of which a person evaluates the quality of their life based on their own unique and subjective criteria [15]. Satisfaction is usually dependent on the distance between one’s expectations and actual results [16].

Adaptation to the situation related to the illness and the treatment process will depend on objective factors as well as subjective mental factors, where the assessment of the situation is dependent on circumstances such as individual experiences from the past, personality traits, or ability to deal with stress, which are of key importance [17,18].

Due to the above, reactions to information concerning the illness are very diverse and may change during the treatment [19]. Adaptation to an illness is, therefore, not a one-time occurrence but a process that leads to restoring balance in the patient’s current situation and eliminating emotional discomfort [20]. Mentalization may be a key component of this adaptation process that influences the patient’s thinking about their illness. Research shows that the ability to mentalize mediates a relationship between experiencing and coping with stress in healthy people, meaning that mentalization is a crucial intermediate mental resource [21]. Adapting to the illness may also result in achieving life satisfaction.

Based on the above, the following purpose of the study was formulated: Is mentalization related to life satisfaction and the adaptation to cancer in female patients diagnosed with cancer?

## 2. Materials and Methods

### 2.1. Study Design

The study was based on the cross-sectional design. The protocol was approved by the Ethics Committee at the Institute of Psychology at University of Gdańsk (No.: 11/2017). Participation in the study was voluntary, and every person had the right to refuse at any point. All participants provided informed consent and privacy policies were followed.

### 2.2. Participants and Setting

The study involved women diagnosed with breast cancer who completed their treatment and participated in a cancer rehabilitation program at the Oncological Centre in Gdansk (Poland). Inclusion criteria were as follows: female, native Polish speaker, diagnosis of breast cancer according to ICD-10 criteria (code C50), and being qualified as a participant in a cancer rehabilitation program. Exclusion criteria included: very poor state of health and women younger than 18 years old.

### 2.3. Data Collection and Outcome Measures

Participant recruitment and data collection were performed simultaneously. Data were collected in person (by self-administration) between March 2018 and September 2018. Three questionnaires were used in the study.

#### 2.3.1. Satisfaction with Life Scale—SWLS

The instrument includes five items [22]. The respondent indicates on a seven-point scale (from “strongly disagree” to “strongly agree”) their agreement with each statement, and the total score indicates their level of satisfaction with life. The possible range of scores is 5 to 35 points. The higher the score, the higher the satisfaction with life. The internal consistency of the scale measured using Cronbach’s alpha in the study group is high and amounts to 0.82; thus, the instrument has good psychometric properties.

#### 2.3.2. Mini-Mental Adjustment to Cancer Scale—Mini-MAC

The Mini-Mental Adjustment to Cancer Scale—Mini-Mac includes 29 statements and assesses four coping responses:

- Anxious preoccupation (anxiety related to illness, perceiving illness as an anxiety-inducing and dangerous phenomenon);
- Fighting spirit (treating the illness as a challenge and taking active action);
- Helplessness–hopelessness (feeling of being lost and powerless);
- Positive redefinition (changing one’s attitude to life and finding value in it).

The instrument is used primarily to measure strategies for coping with cancer, defined as the ability to adapt to cancer [23]. It may also be used to assess the respondents’ reactions of the respondents to a cancer diagnosis and track changes resulting from the treatment or rehabilitation process. The respondent indicates on a four-point scale (where 1 means “definitely not”, and 4 means “definitely yes”) whether a given statement applies to them at a given moment. The higher the result for a given strategy, the stronger the behavior typical for a given strategy of coping with cancer. The instrument has good psychometric properties in the study group; the internal consistency of the scale measured using Cronbach’s alpha in the study group for each scale was as follows: AP: 0.71, FS: 0.70, HH: 0.72, and PR: 0.63.

#### 2.3.3. The Mentalization Questionnaire (MZQ)

The Mentalization Questionnaire (MZQ) by Hausberg et al. [24] is an instrument with 15 items divided into four subscales (refusing self-reflection, emotional awareness, psychic equivalence mode, regulation of affect). On a five-point Likert-style scale (where 1 means “I disagree”, and 5 means “I agree”), respondents specify the degree to which they agree with a given statement. The total score is calculated by summing up the points scored for each question. The maximum score is 75. The lower the total score, the higher the respondent’s ability to mentalize. The instrument has good psychometric properties in the study group; Cronbach’s alpha in the study group was 0.82.

### 2.4. Statistical Analysis

Data analysis was performed with usage of the SPSS program, version 27 (IBM SPSS Statistics; Predictive Solutions Sp. z o.o., Krakow, Poland). The first step in the analysis of the descriptive results of the study was to calculate the average values and standard deviations of the examined variables and determine the normal distribution of examined data. Pearson correlation coefficients between the values were then calculated, and a linear regression analysis was carried out.

## 3. Results

The study was performed among 41 women over 18 years old (*M* = 59.88; *SD* = 8.81). Most participants were undergoing radical treatment (92.7%) and married (65.8%), while 46.3% declared secondary education level and 41.5% tertiary education. More social and demographic details are included in Table 1.

The distribution of the analyzed variables is close to normal distributions, enabling the use of parametric tests. The average level of mentalization in the examined group was 45.51 points, indicating an average intensity of the variable among the study group. Life satisfaction was at a moderate level. Regarding the level of mental adjustment to cancer, the highest average results were obtained for the two positive strategies (fighting spirit and positive redefinition) (see Table 2).

The next stage of the analysis involved plotting correlations between mentalization, life satisfaction, and mental adjustment to the illness. The calculated correlation coefficients are shown in Table 3.

The calculated values of the Pearson correlation coefficient indicate the presence of a correlation between mentalization (both its general value and individual dimensions) and satisfaction with life/mental adjustment to cancer. The general value of mentalization positively although with moderate intensity correlates with anxious preoccupation, and helplessness–hopelessness although negatively and with moderate intensity correlates with satisfaction with life measured using the SWLS questionnaire.

Analyses indicate the presence of significant correlations between individual mentalization factors and the analyzed dimensions of mental adjustment to illness and satisfaction with life. The strongest positive correlations were observed between refusing self-reflection and helplessness–hopelessness and anxious preoccupation. Moderate positive correlations were observed between emotional awareness and anxious preoccupation and helplessness–hopelessness. Moderate positive correlations were observed between psychic equivalence, regulation of affect, and anxious preoccupation. Moderate negative correlations were observed between refusing self-reflection and satisfaction with life and positive redefinition, whereas the weakest negative correlation was observed between regulation of affect and fighting spirit.

To finalize the statistical analyses, an attempt was made to answer whether the general score for mentalization and its dimensions help predict the level of satisfaction with life and mental adjustment to the illness. To this end, a linear regression analysis was performed using the entry method. We first looked for predictors of satisfaction with life and then for coping strategies. The results of the analysis are shown in Table 4, Table 5, Table 6, Table 7, Table 8, Table 9, Table 10, Table 11, Table 12 and Table 13.

Mentalization was a predictor of satisfaction with life. Based on the value of coefficient B, we can conclude that the higher the score of mentalization (lower level of mentalization of the respondent), the lower the level of satisfaction with life.

**Table 5:** Predictors of satisfaction with life.

	β	B	SE	*t*	*p*
Refusing self-reflection	−0.548	−1.212	−1.212	−3.495	0.001
Fixed value		32.893	6.518	5.047	0.000

R = 0.57; R^2^ = 0.33. Annotation: β, the standardized beta; B, unstandardized beta; SE, standard error; *p*, significant; *t*, the test statistic.

One of the dimensions of mentalization—refusing self-reflection—turned out to be a predictor of satisfaction with life. Based on the value of coefficient B, we can conclude that the higher the intensity of refusing self-reflection (stronger value of refusing self-reflection), the lower the level of satisfaction with life in respondents.

**Table 6:** Predictors of anxious preoccupation.

	β	B	SE	*t*	*p*
Mentalization—General score	0.867	0.774	0.071	10.858	0.000
Fixed value	0.867	0.774	0.071	10.858	0.000

R = 0.86; R^2^ = 0.75. Annotation: β, the standardized beta; B, unstandardized beta; SE, standard error; *p*, significant; *t*, the test statistic.

Mentalization proved to be a predictor of anxious preoccupation. Based on the value of coefficient B, we can conclude that the higher the intensity of mentalization (lower value of mentalization), the higher the level of anxious preoccupation.

**Table 7:** Predictors of anxious preoccupation.

	β	B	SE	*t*	*p*
Refusing self-reflection	0.552	0.923	0.155	5.945	0.000
Psychic equivalence	0.209	0.547	0.235	2.322	0.026
Regulation of affect	0.354	0.823	0.194	4.239	0.000
Fixed value		−9.026	2.919	−3.093	0.004

R = 0.87; R^2^ = 0.76. Annotation: β, the standardized beta; B, unstandardized beta; SE, standard error; *p*, significant; *t*, the test statistic.

Refusing self-reflection, regulation of affect, and psychic equivalence proved to be a predictor of anxious preoccupation. Based on the value of coefficient B, we can conclude that the higher the intensity of the three above dimensions of mentalization (higher value of refusing self-reflection, lower psychic stability, and lower ability to regulate affect), the higher the level of anxious preoccupation.

**Table 8:** Predictors of fighting spirit.

	β	B	SE	*t*	*p*
Mentalization—General score	−0.129	−0.069	0.085	−0.813	0.421
Fixed value		27.200	2.757	9.865	0.000

R = 0.12; R^2^ = 0.01. Annotation: β, the standardized beta; B, unstandardized beta; SE, standard error; *p*, significant; *t*, the test statistic.

The general mentalization score proved not to be a predictor of the fighting spirit strategy.

**Table 9:** Predictors of fighting spirit.

	β	B	SE	*t*	*p*
Regulation of affect	−0.365	−0.809	0.208	−2.445	0.019
Fixed value		29.051	1.700	17.087	0.000

R = 0.26; R^2^ = 0.13. Annotation: β, the standardized beta; B, unstandardized beta; SE, standard error; *p*, significant; *t*, the test statistic.

Regulation of affect proved to be a predictor of fighting spirit. Based on the value of coefficient B, we can conclude that the lower the intensity of regulation of affect (higher ability to regulate affect), the higher the intensity of the fighting spirit strategy.

**Table 10:** Predictors of helplessness–hopelessness.

	β	B	SE	*t*	*p*
Mentalization—General score	0.691	0.525	0.088	5.975	0.000
Fixed value		−6.392	2.849	−2.244	0.031

R = 0.69; R^2^ = 0.47. Annotation: β, the standardized beta; B, unstandardized beta; SE, standard error; *p*, significant; *t*, the test statistic.

Mentalization of affect proved to be a predictor of helplessness–hopelessness. Based on the value of coefficient B, we can conclude that the higher the intensity of mentalization (lower ability to mentalize), the higher the level of helplessness–hopelessness.

**Table 11:** Predictors of helplessness–hopelessness.

	β	B	SE	*t*	*p*
Refusing self-reflection	0.683	0.972	0.163	5.961	0.456
Emotional awareness	0.261	0.726	0.322	2.252	0.096
Psychic equivalence	−0.085	−0.189	0.247	−0.763	0.451
Regulation of affect	0.049	0.097	0.204	0.476	0.637
Fixed value		−0.024	3.065	−0.008	0.994

R = 0.80; R^2^ = 0.64. Annotation: β, the standardized beta; B, unstandardized beta; SE, standard error; *p*, significant; *t*, the test statistic.

Individual dimensions of mentalization proved not to be a predictor of the helplessness–hopelessness strategy.

**Table 12:** Predictors of positive redefinition.

	β	B	SE	*t*	*p*
Mentalization—General score	−0.135	−0.086	0.100	−0.852	0.400
Fixed value		25.675	3.254	7.890	0.000

R = 0.13; R^2^ = 0.01. Annotation: β, the standardized beta; B, unstandardized beta; SE, standard error; *p*, significant; *t*, the test statistic.

The general mentalization score proved not to be a predictor of positive redefinition.

**Table 13:** Predictors of positive redefinition.

	β	B	SE	*t*	*p*
Refusing self-reflection	−0.475	−0.563	0.195	−2.891	0.006
Psychic equivalence	−0.410	0.761	0.295	2.577	0.014
Fixed value		18.918	3.663	5.165	0.000

R = 0.51; R^2^ = 0.26. Annotation: β, the standardized beta; B, unstandardized beta; SE, standard error; *p*, significant; *t*, the test statistic.

Refusing self-reflection and psychic equivalence proved to be predictors of positive redefinition. Based on the value of coefficient B, we can conclude that the higher the intensity of refusing self-reflection (stronger refusal) and the lower the psychic equivalence (greater psychic stability), the lower the level of positive redefinition.

## 4. Discussion

To the authors’ knowledge, no prior research on mentalization in patients with cancer has been carried out. The hypothesis that mentalization among female patients dealing with breast cancer is related to satisfaction with life and mental adjustment to the illness has been confirmed.

The women in the study group, who were breast cancer patients treated at a hospital oncological rehabilitation ward, declared an average level of mentalization. The evaluation of life satisfaction among the women in the study group is also around the average level, which means that the study did not demonstrate a reduced life satisfaction among the respondents. Prior research conducted by Kozaka and Kobus [25] indicated a similar level of satisfaction with life among cancer patients. The average level of satisfaction with life indicates that cancer must not necessarily result in negative consequences as the stereotype would have us believe.

A cancer diagnosis may lead to positive changes related to the so-called “benefit finding” [26]. This process may lead to post-traumatic growth, where a person battling cancer starts to reassess their entire life, increasing their sense of meaning in life and responsibility for it. The entire process of thinking about the illness (mentalization) may lead to personal development and growth [27]. Other research indicates that as many as 76% of patients benefit from their battle with their illness [28]. An improved capacity for mentalization and one of its dimensions (i.e., refusing self-reflection) was correlated with improved satisfaction with life in our study.

This theory is also confirmed by other results of our study in terms of positive redefinition and fighting spirit. Results of the study indicate a moderate correlation between mentalization and satisfaction with life and mental adjustment to the illness among the respondents. Furthermore, the general mentalization score and one of its dimensions (refusing self-reflection) proved to be a predictor of satisfaction with life. That means those who accept their emotional states, reflect on them, deliberate on what they feel, and try to understand the situation that they are in without rejecting it may feel much more satisfied with their life. Among the strategies of coping with and adapting to cancer, there were significant positive correlations between mentalization with increased use of anxious preoccupation and helplessness–hopelessness strategies. The lower the level of mentalization, the more frequent the use of destructive coping strategies. Those characterized by non-transparent emotional states do not make an effort to understand their emotional states and intentions and more frequently engage in destructive coping mechanisms. Such patients experience greater anxiety related to their illness. They begin to feel helpless and perceive their illness as a threat rather than a challenge, which in turn may lead to increased stress and reduced motivation to battle the illness.

The general mentalization score and its three dimensions (refusing self-reflection, psychic equivalence, and regulation of affect) proved to be predictors of anxious preoccupation. The tendency to use the anxious preoccupation strategy increases with the reduction of declared intensity in the three above dimensions. Such people experience increased anxiety and concern and feel like they are no longer in control of their own lives. Previous studies show that mentalization improves mental health condition and reduces maladaptive defense mechanisms, which may explain the relationship between better-developed mentalization ability and also stress reduction in chronically ill patients [29].

Certain dimensions of mentalization proved to be predictors of the use of constructive strategies for adjusting to the illness; refusing self-reflection was a predictor of using the fighting spirit strategy, whereas psychic equivalence was a predictor of the positive redefinition strategy. The results gained in all predictions indicated an upward correlation. The higher the respondent’s ability to regulate their affect and reflect on their internal emotions by relating them to their illness and current situation, the more frequent their use of constructive adjustment strategies.

The results of the study may be valuable for scientists and clinical psychologists working directly with cancer patients. Encouraging patients to mentalize (developing their mentalization skills as part of their treatment) and to use constructive adjustment strategies appear to be valid for their mental health. That would enable cancer patients to achieve a higher level of satisfaction with life, which might lead to greater involvement in the treatment process and ultimately improve their prognoses.

### 4.1. Strengths of the Study

The purpose of the proposed study was to explore psychological predictors linked to higher satisfaction with life in cancer patients. The study proved that the ability to mentalize, which had previously not been verified as a potential protective factor in cancer patients, is an important mental resource related to satisfaction with life. That is an important theoretical guideline for practicing counsellors who are tasked with improving the mental health of cancer patients.

### 4.2. Limitations and Weaknesses of the Study

The results of the study should be treated with a degree of caution. The study is exploratory; therefore, it would be advisable to continue research on the above-described issue, in particular among cancer patients. The ability to generalize the results of the study is limited due to the low sample size and the fact that the study was performed on patients suffering from a single type of cancer. This is a cross-sectional study, which does not allow to make causal inferences. Furthermore, more factors than those included in this study should be considered (including the complexity and stage of cancer and the medication taken by patients).

### 4.3. Future Directions

In the next step, it would be valuable to assess coping strategies and their relationship to disease severity and mentalization. Furthermore, the other types of cancer should be considered in further studies. Moreover, it is interesting to examine the effect of potential programs supporting mentalization in patients diagnosed with oncological disease on their ability to cope.

## 5. Conclusions

Mentalization was related to life satisfaction and adjustment to cancer in patients with breast cancer, which is in line with previous studies suggesting the crucial role of subjective psychological factors in maintaining mental health.

## Figures and Tables

**Table 1:** Social and demographic characteristics of the study group.

Social and Demographic Characteristics	Subjects	
	*n*	%
**Marital status**		
Married	27	65.8
Single	2	4.9
Divorced	3	7.3
Separated	3	7.3
Widowed	6	14.7
**Education level**		
Vocational	5	12.2
Secondary	19	46.3
Tertiary	17	41.5
**Professional status**		
Actively employed	17	41.5
Unemployed	2	4.9
Retired	19	46.3
On disability benefits	3	7.3
**Treatment**		
Radical	38	92.7
Palliative	3	7.3

Annotation: *n*, number of respondents; %, percentage of respondents.

**Table 2:** Average values and standard deviation of life satisfaction, mental adjustment, and mentalization.

Analyzed Variables	*M*	*SD*
**Mentalization—General score**	45.51	11.65
Refusing self-reflection	7.09	2.26
Emotional awareness	6.60	1.15
Psychic equivalence mode	10.41	1.44
Regulation of affect	8.00	1.62
**Satisfaction with life**	21.36	5.01
**Anxious preoccupation**	13.87	3.78
**Fighting spirit**	24.97	2.27
**Helplessness–hopelessness**	10.48	3.22
**Positive redefinition**	22.92	2.68

Annotation: *M*, mean; *SD*, standard deviation.

**Table 3:** Coefficients of correlation between satisfaction with life, mental adjustment, and mentalization.

Mentalization	Satisfaction with Life	Anxious Preoccupation	Fighting Spirit	Helplessness Hopelessness	Positive Redefinition
Mentalization—General score	−0.59 **	0.41 **	−0.23	0.34 *	−0.22
Refusing self-reflection	−0.57 **	0.76 **	−0.16	0.77 **	−0.39 *
Emotional awareness	−0.22	0.47 **	0.01	0.50 **	−0.10
Psychic equivalence	−0.26	0.47 **	0.27	0.25	0.23
Regulation of affect	−0.14	0.40 **	−0.36 *	0.15	0.00

** Correlation significance *p* < 0.01; * correlation significance *p* < 0.05.

**Table 4:** Predictors of satisfaction with life.

	β	B	SE	*t*	*p*
Mentalization—General score	−0.504	−0.595	0.163	−3.643	0.001
Fixed value		40.487	5.294	7.648	0.000

R = 0.50; R^2^ = 0.25. Annotation: β, the standardized beta; B, unstandardized beta; SE, standard error; *p*, significant; *t*, the test statistic.

## Data Availability

The data that support the findings of this study are available on request from the corresponding author (M.C., J.K.).
